# Supplementary material for: Informal sector employment and the health outcomes of older workers in India
Source: PLoS One. 2023 Feb 22;18(2):e0266576. doi: 10.1371/journal.pone.0266576 (PMC9946227; doi:10.1371/journal.pone.0266576)
Supplement: S1 Appendix — (DOCX) [file pone.0266576.s001.docx]

1. **Chronic Health Conditions**

**Table-S1: Result of logistic regression for CHC by gender**

| **Covariates** | **Odds Ratio** | |
| --- | --- | --- |
|  | **Male** | **Female** |
| **Type of work** |  |  |
| Formal® |  |  |
| Informal | 0.864** (0.772 0.968) | 0.954 (0.791 1.150) |
| ***Socio-economic & demographic*** |  |  |
| **Age groups** |  |  |
| 60-65® |  |  |
| 65+ | 1.450**** (1.303 1.612) | 1.286*** (1.098 1.506) |
| **Caste groups** |  |  |
| General® |  |  |
| Scheduled Tribe | 0.527**** (0.434 0.639) | 0.688** (0.518 0.914) |
| Scheduled Caste | 0.803*** (0.68 0.948) | 1.196 (0.931 1.536) |
| Other Backward Class | 0.847** (0.742 0.967) | 1.08 (0.863 1.353) |
| **Religion** |  |  |
| Hindu® |  |  |
| Muslim | 1.217** (1.033 1.434) | 1.705*** (1.243 2.337) |
| Others | 1.187 (0.989 1.425) | 1.309** (1.009 1.698) |
| **Education level** |  |  |
| Low® |  |  |
| Middle | 1.162** (1.02 1.324) | 1.036 (0.701 1.533) |
| High | 1.249** (1.011 1.542) | 1.649 (0.817 3.329) |
| **Marital status** |  |  |
| Currently married® |  |  |
| Others | 0.896 (0.769 1.043) | 1.182** (1.013 1.379) |
| **Place of residence** |  |  |
| Rural® |  |  |
| Urban | 1.759**** (1.547 1.999) | 1.643**** (1.348 2.004) |
| **Wealth** |  |  |
| Low® |  |  |
| Medium | 1.359**** (1.198 1.542) | 1.438**** (1.201 1.722) |
| High | 1.714**** (1.482 1.983) | 1.892**** (1.51 2.372) |
| **Household size** | 0.924** (0.869 0.982) | 0.947 (0.879 1.019) |
| ***Work Characteristics*** |  |  |
| **Working hours** |  |  |
| Less than 24 hours® |  |  |
| 24-48 hours | 0.911 (0.804 1.032) | 0.924 (0.777 1.099) |
| 48+ hours | 0.805*** (0.703 0.923) | 0.886 (0.718 1.093) |
| **Ln(Wage)** | 1.044 (0.982 1.11) | 0.947 (0.866 1.035) |
| **Duration being in current work** |  |  |
| Less than 15 years® |  |  |
| 15-30 years | 1.008 (0.854 1.191) | 0.908 (0.711 1.16) |
| 30-45 years | 0.899 (0.776 1.041) | 0.903 (0.727 1.122) |
| 45 years and over | 0.914 (0.785 1.063) | 0.841 (0.677 1.044) |
| ***Life style behaviour*** |  |  |
| **Drinking Alcohol** |  |  |
| No® |  |  |
| Yes | 0.959 (0.839 1.096) | 0.800 (0.559 1.145) |
| **Smoking/Consuming Tobacco** |  |  |
| No® |  |  |
| Yes | 1.035 (0.927 1.155) | 0.867 (0.731 1.028) |
| ***Physical Activity*** |  |  |
| **Vigorous** |  |  |
| Never® |  |  |
| Rare | 0.740**** (0.641 0.854) | 0.744*** (0.603 0.917) |
| Everyday | 0.668**** (0.59 0.757) | 0.951 (0.788 1.148) |
| **Moderate** |  |  |
| Never® |  |  |
| Rare | 0.994 (0.85 1.162) | 1.164 (0.879 1.543) |
| Everyday | 0.979 (0.864 1.109) | 0.885 (0.716 1.095) |
| **Yoga/Pranayam** |  |  |
| Never® |  |  |
| Rare | 1.317** (1.045 1.66) | 0.965 (0.644 1.448) |
| Everyday | 1.111 (0.942 1.312) | 1.686*** (1.242 2.288) |
| **Childhood health** |  |  |
| Good/Fair® |  |  |
| Poor | 1.544*** (1.153 2.068) | 2.734*** (1.517 4.926) |
| **Regions** |  |  |
| North® |  |  |
| Central | 0.507**** (0.417 0.616) | 0.538**** (0.39 0.741) |
| East | 0.885 (0.743 1.054) | 0.871 (0.648 1.171) |
| Northeast | 0.653**** (0.523 0.816) | 0.617*** (0.436 0.874) |
| West | 1.064 (0.866 1.309) | 1.247 (0.929 1.673) |
| South | 1.401**** (1.17 1.677) | 1.349** (1.02 1.785) |
| Union Territories | 1.191 (0.938 1.514) | 0.927 (0.648 1.326) |
| **Constant** | **0.765** | **1.162** |

**Note: ® reference category, ****P<0.001, ***P<0.01, **P<0.05**

**Table-S2: Result of logistic regression for CHC by place of residence**

| **Covariates** | **Odds Ratio** | |
| --- | --- | --- |
|  | **Rural** | **Urban** |
| **Type of work** |  |  |
| Formal® |  |  |
| Informal | 0.896 (0.799 1.006) | 0.871 (0.727 1.044) |
| ***Socio-economic & demographic*** |  |  |
| **Gender** |  |  |
| Male® |  |  |
| Female | 1.193*** (1.047 1.358) | 0.985 (0.775 1.252) |
| **Age groups** |  |  |
| 60-65® |  |  |
| 65+ | 1.353**** (1.221 1.500) | 1.526**** (1.286 1.810) |
| **Caste groups** |  |  |
| General® |  |  |
| Scheduled Tribe | 0.563**** (0.468 0.676) | 0.683** (0.482 0.967) |
| Scheduled Caste | 0.89 (0.757 1.046) | 0.980 (0.748 1.284) |
| Other Backward Class | 0.936 (0.815 1.075) | 0.852 (0.691 1.051) |
| **Religion** |  |  |
| Hindu® |  |  |
| Muslim | 1.381*** (1.149 1.66) | 1.269** (1.000 1.611) |
| Others | 1.277*** (1.077 1.515) | 1.178 (0.869 1.596) |
| **Education level** |  |  |
| Low® |  |  |
| Middle | 1.28*** (1.101 1.489) | 0.973 (0.788 1.202) |
| High | 1.198 (0.901 1.593) | 1.284 (0.954 1.727) |
| **Marital status** |  |  |
| Currently married® |  |  |
| Others | 1.034 (0.914 1.170) | 0.934 (0.753 1.159) |
| **Wealth** |  |  |
| Low® |  |  |
| Medium | 1.334**** (1.181 1.507) | 1.483**** (1.218 1.806) |
| High | 1.678**** (1.454 1.937) | 1.867**** (1.468 2.374) |
| **Household size** | 0.932** (0.882 0.984) | 0.946 (0.863 1.037) |
| ***Work Characteristics*** |  |  |
| **Working hours** |  |  |
| Less than 24 hours® |  |  |
| 24-48 hours | 0.901 (0.802 1.013) | 0.922 (0.75 1.133) |
| 48+ hours | 0.812*** (0.71 0.928) | 0.861 (0.69 1.073) |
| **Ln(Wage)** | 1.002 (0.944 1.063) | 1.034 (0.94 1.138) |
| **Duration being in current work** |  |  |
| Less than 15 years® |  |  |
| 15-30 years | 1.053 (0.887 1.25) | 0.863 (0.688 1.082) |
| 30-45 years | 0.836** (0.723 0.968) | 1.030 (0.825 1.286) |
| 45 years and over | 0.828** (0.717 0.957) | 1.102 (0.848 1.431) |
| ***Life style behaviour*** |  |  |
| **Drinking Alcohol** |  |  |
| No® |  |  |
| Yes | 0.951 (0.827 1.094) | 0.945 (0.726 1.230) |
| **Smoking/Consuming Tobacco** |  |  |
| No® |  |  |
| Yes | 1.058 (0.95 1.178) | 0.788** (0.658 0.944) |
| ***Physical Activity*** |  |  |
| **Vigorous** |  |  |
| Never® |  |  |
| Rare | 0.746**** (0.652 0.853) | 0.756** (0.586 0.975) |
| Everyday | 0.744**** (0.658 0.841) | 0.766*** (0.627 0.935) |
| **Moderate** |  |  |
| Never® |  |  |
| Rare | 1.041 (0.888 1.221) | 0.995 (0.766 1.292) |
| Everyday | 0.933 (0.821 1.059) | 0.973 (0.797 1.188) |
| **Yoga/Pranayam** |  |  |
| Never® |  |  |
| Rare | 1.296** (1.022 1.642) | 1.124 (0.778 1.624) |
| Everyday | 1.297*** (1.091 1.542) | 1.047 (0.802 1.366) |
| **Childhood health** |  |  |
| Good/Fair® |  |  |
| Poor | 1.784**** (1.328 2.398) | 1.522 (0.884 2.618) |
| **Regions** |  |  |
| North® |  |  |
| Central | 0.455**** (0.377 0.549) | 0.78 (0.549 1.108) |
| East | 0.771*** (0.651 0.914) | 1.395** (1.001 1.945) |
| Northeast | 0.585**** (0.474 0.723) | 0.734 (0.495 1.089) |
| West | 1.178 (0.966 1.436) | 1.162 (0.848 1.591) |
| South | 1.362**** (1.146 1.62) | 1.525*** (1.126 2.066) |
| Union Territories | 0.951 (0.728 1.241) | 1.309 (0.949 1.806) |
| **Constant** | **1.002** | **1.093** |

**Note: ® reference category, ****P<0.001, ***P<0.01, **P<0.05**

**Table-S3: Result of logistic regression for CHC by age-groups**

| **Covariates** | **Odds Ratio** | |
| --- | --- | --- |
|  | **60-65** | **65+** |
| **Type of work** |  |  |
| Formal® |  |  |
| Informal | 0.854** (0.75 0.973) | 0.926 (0.802 1.070) |
| ***Socio-economic & demographic*** |  |  |
| **Gender** |  |  |
| Male® |  |  |
| Female | 1.159 (0.997 1.348) | 1.100 (0.925 1.309) |
| **Caste groups** |  |  |
| General® |  |  |
| Scheduled Tribe | 0.597**** (0.483 0.737) | 0.532**** (0.42 0.675) |
| Scheduled Caste | 0.895 (0.745 1.075) | 0.906 (0.738 1.114) |
| Other Backward Class | 0.909 (0.78 1.06) | 0.883 (0.745 1.048) |
| **Religion** |  |  |
| Hindu® |  |  |
| Muslim | 1.338*** (1.1 1.626) | 1.328** (1.072 1.646) |
| Others | 1.211 (0.988 1.485) | 1.272** (1.024 1.581) |
| **Education level** |  |  |
| Low® |  |  |
| Middle | 1.192** (1.013 1.402) | 1.119 (0.928 1.35) |
| High | 1.267 (0.978 1.642) | 1.328 (0.97 1.818) |
| **Place of residence** |  |  |
| Rural® |  |  |
| Urban | 1.64**** (1.42 1.894) | 1.856**** (1.582 2.178) |
| **Marital status** |  |  |
| Currently married® |  |  |
| Others | 1.094 (0.942 1.271) | 0.940 (0.807 1.095) |
| **Wealth** |  |  |
| Low® |  |  |
| Medium | 1.444**** (1.259 1.657) | 1.322**** (1.132 1.544) |
| High | 1.761**** (1.495 2.073) | 1.789**** (1.489 2.148) |
| **Household size** | 0.953 (0.893 1.018) | 0.908*** (0.849 0.972) |
| ***Work Characteristics*** |  |  |
| **Working hours** |  |  |
| Less than 24 hours® |  |  |
| 24-48 hours | 0.865** (0.753 0.994) | 0.97 (0.838 1.124) |
| 48+ hours | 0.796*** (0.683 0.927) | 0.875 (0.736 1.039) |
| **Ln(Wage)** | 1.044 (0.975 1.118) | 0.982 (0.912 1.058) |
| **Duration being in current work** |  |  |
| Less than 15 years® |  |  |
| 15-30 years | 1.008 (0.843 1.206) | 0.94 (0.759 1.165) |
| 30-45 years | 0.95 (0.813 1.11) | 0.834 (0.685 1.015) |
| 45 years and over | 0.939 (0.785 1.122) | 0.838** (0.703 0.999) |
| ***Life style behaviour*** |  |  |
| **Drinking Alcohol** |  |  |
| No® |  |  |
| Yes | 0.952 (0.81 1.12) | 0.941 (0.779 1.137) |
| **Smoking/Consuming Tobacco** |  |  |
| No® |  |  |
| Yes | 0.974 (0.86 1.103) | 0.988 (0.863 1.132) |
| ***Physical Activity*** |  |  |
| **Vigorous** |  |  |
| Never® |  |  |
| Rare | 0.764*** (0.652 0.896) | 0.723**** (0.607 0.861) |
| Everyday | 0.744**** (0.648 0.855) | 0.76*** (0.651 0.887) |
| **Moderate** |  |  |
| Never® |  |  |
| Rare | 1.071 (0.889 1.29) | 0.984 (0.807 1.2) |
| Everyday | 1.025 (0.885 1.186) | 0.848** (0.726 0.99) |
| **Yoga/Pranayam** |  |  |
| Never® |  |  |
| Rare | 1.035 (0.806 1.33) | 1.704*** (1.211 2.397) |
| Everyday | 1.258** (1.032 1.534) | 1.19 (0.961 1.473) |
| **Childhood health** |  |  |
| Good/Fair® |  |  |
| Poor | 1.522** (1.084 2.139) | 1.987*** (1.317 2.999) |
| **Regions** |  |  |
| North® |  |  |
| Central | 0.537**** (0.432 0.667) | 0.498**** (0.386 0.642) |
| East | 0.902 (0.74 1.1) | 0.868 (0.69 1.092) |
| Northeast | 0.667*** (0.519 0.858) | 0.621*** (0.471 0.818) |
| West | 1.172 (0.937 1.466) | 1.085 (0.844 1.395) |
| South | 1.45**** (1.186 1.773) | 1.331** (1.061 1.669) |
| Union Territories | 1.084 (0.832 1.413) | 1.087 (0.808 1.462) |
| **Constant** | **0.571** | **1.872** |

**Note: ® reference category, ****P<0.001, ***P<0.01, **P<0.05**

1. **Functional Limitations**

**Table-S4: Result of logistic regression for FL by gender**

| **Covariates** | **Odds Ratio** | |
| --- | --- | --- |
|  | **Male** | **Female** |
| **Res_CHC** | 1.536**** (1.248 1.890) | 1.539** (1.095 2.165) |
| **Type of work** |  |  |
| Formal® |  |  |
| Informal | 0.969 (0.860 1.091) | 0.755*** (0.628 0.907) |
| **Type of work*Res_CHC** | 1.032 (0.809 1.316) | 0.918 (0.629 1.340) |
| ***Socio-economic & demographic*** |  |  |
| **Age groups** |  |  |
| 60-65® |  |  |
| 65+ | 1.396**** (1.250 1.559) | 1.513**** (1.296 1.767) |
| **Caste groups** |  |  |
| General® |  |  |
| Scheduled Tribe | 0.782** (0.640 0.954) | 0.834 (0.633 1.098) |
| Scheduled Caste | 0.885 (0.742 1.055) | 0.935 (0.728 1.200) |
| Other Backward Class | 0.904 (0.784 1.042) | 1.018 (0.813 1.275) |
| **Religion** |  |  |
| Hindu® |  |  |
| Muslim | 1.243** (1.049 1.473) | 1.037 (0.757 1.420) |
| Others | 0.863 (0.711 1.049) | 0.735** (0.571 0.944) |
| **Education level** |  |  |
| Low® |  |  |
| Middle | 0.632**** (0.547 0.730) | 0.695 (0.465 1.039) |
| High | 0.512**** (0.398 0.659) | 0.527 (0.272 1.021) |
| **Marital status** |  |  |
| Currently married® |  |  |
| Others | 1.419**** (1.219 1.652) | 1.169** (1.005 1.359) |
| **Place of residence** |  |  |
| Rural® |  |  |
| Urban | 0.642**** (0.557 0.739) | 0.671**** (0.55 0.818) |
| **Wealth** |  |  |
| Low® |  |  |
| Medium | 0.784**** (0.689 0.892) | 0.833** (0.698 0.993) |
| High | 0.803*** (0.69 0.936) | 0.891 (0.713 1.113) |
| **Household size** | 1.065 (0.999 1.134) | 1.021 (0.95 1.098) |
| ***Work Characteristics*** |  |  |
| **Working hours** |  |  |
| Less than 24 hours® |  |  |
| 24-48 hours | 1.033 (0.907 1.176) | 0.989 (0.835 1.172) |
| 48+ hours | 0.966 (0.837 1.114) | 0.935 (0.763 1.147) |
| **Ln(Wage)** | 0.875**** (0.82 0.933) | 0.942 (0.864 1.028) |
| **Duration being in current work** |  |  |
| Less than 15 years® |  |  |
| 15-30 years | 1.044 (0.874 1.247) | 0.995 (0.781 1.268) |
| 30-45 years | 0.971 (0.83 1.136) | 1.059 (0.855 1.312) |
| 45 years and over | 1.11 (0.949 1.3) | 1.058 (0.854 1.311) |
| ***Life style behaviour*** |  |  |
| **Drinking Alcohol** |  |  |
| No® |  |  |
| Yes | 1.190** (1.04 1.363) | 1.334 (0.96 1.854) |
| **Smoking/Consuming Tobacco** |  |  |
| No® |  |  |
| Yes | 1.276**** (1.136 1.435) | 1.282*** (1.085 1.514) |
| ***Physical Activity*** |  |  |
| **Vigorous** |  |  |
| Never® |  |  |
| Rare | 0.954 (0.822 1.108) | 0.954 (0.779 1.169) |
| Everyday | 0.817*** (0.716 0.933) | 0.974 (0.809 1.172) |
| **Moderate** |  |  |
| Never® |  |  |
| Rare | 1.066 (0.908 1.253) | 1.154 (0.873 1.527) |
| Everyday | 0.915 (0.802 1.043) | 0.996 (0.806 1.229) |
| **Yoga/Pranayam** |  |  |
| Never® |  |  |
| Rare | 1.001 (0.779 1.286) | 1.372 (0.929 2.025) |
| Everyday | 0.987 (0.823 1.183) | 0.986 (0.730 1.333) |
| **Childhood health** |  |  |
| Good/Fair® |  |  |
| Poor | 1.318 (0.978 1.775) | 1.525 (0.867 2.684) |
| ***Regions*** |  |  |
| North® |  |  |
| Central | 0.988 (0.804 1.214) | 1.199 (0.884 1.628) |
| East | 1.173 (0.972 1.416) | 1.696**** (1.267 2.27) |
| Northeast | 0.862 (0.675 1.1) | 1.026 (0.73 1.443) |
| West | 1.644**** (1.319 2.047) | 2.387**** (1.777 3.207) |
| South | 1.927**** (1.591 2.334) | 2.310**** (1.745 3.059) |
| Union Territories | 1.159 (0.89 1.51) | 1.602*** (1.13 2.273) |
| **Constant** | **1.153** | **1.135** |

**Note: ® reference category, ****P<0.001, ***P<0.01, **P<0.05**

**Table-S5: Result of logistic regression for FL by place of residence**

| **Covariates** | **Odds Ratio** | |
| --- | --- | --- |
|  | **Rural** | **Urban** |
| **Res_CHC** | 1.514**** (1.232 1.861) | 1.558** (1.107 2.193) |
| **Type of work** |  |  |
| Formal® |  |  |
| Informal | 0.921 (0.821 1.034) | 0.847 (0.691 1.039) |
| **Type of work*Res_CHC** | 0.957 (0.757 1.211) | 1.192 (0.789 1.802) |
| ***Socio-economic & demographic*** |  |  |
| **Gender** |  |  |
| Male® |  |  |
| Female | 1.899**** (1.671 2.159) | 2.228**** (1.720 2.885) |
| **Age groups** |  |  |
| 60-65® |  |  |
| 65+ | 1.37**** (1.236 1.518) | 1.700**** (1.408 2.053) |
| **Caste groups** |  |  |
| General® |  |  |
| Scheduled Tribe | 0.790** (0.660 0.947) | 0.704 (0.469 1.056) |
| Scheduled Caste | 0.886 (0.751 1.046) | 0.963 (0.713 1.300) |
| Other Backward Class | 0.914 (0.792 1.054) | 1.033 (0.816 1.308) |
| **Religion** |  |  |
| Hindu® |  |  |
| Muslim | 1.224** (1.015 1.476) | 1.201 (0.930 1.552) |
| Others | 0.833** (0.701 0.990) | 0.865 (0.612 1.222) |
| **Education level** |  |  |
| Low® |  |  |
| Middle | 0.661**** (0.562 0.778) | 0.567**** (0.443 0.726) |
| High | 0.455**** (0.323 0.640) | 0.531**** (0.377 0.748) |
| **Marital status** |  |  |
| Currently married® |  |  |
| Others | 1.328**** (1.178 1.497) | 1.161 (0.920 1.465) |
| **Wealth** |  |  |
| Low® |  |  |
| Medium | 0.773**** (0.686 0.871) | 0.874 (0.701 1.091) |
| High | 0.765**** (0.662 0.883) | 1.061 (0.814 1.383) |
| **Household size** | 1.063** (1.007 1.122) | 1.004 (0.908 1.11) |
| ***Work Characteristics*** |  |  |
| **Working hours** |  |  |
| Less than 24 hours® |  |  |
| 24-48 hours | 1.047 (0.932 1.177) | 0.912 (0.727 1.143) |
| 48+ hours | 0.934 (0.817 1.068) | 0.956 (0.748 1.223) |
| **Ln(Wage)** | 0.936** (0.881 0.993) | 0.804**** (0.724 0.894) |
| **Duration being in current work** |  |  |
| Less than 15 years® |  |  |
| 15-30 years | 1.031 (0.865 1.228) | 1.094 (0.849 1.41) |
| 30-45 years | 1.003 (0.865 1.164) | 1.055 (0.823 1.352) |
| 45 years and over | 1.08 (0.933 1.249) | 1.077 (0.812 1.427) |
| ***Life style behaviour*** |  |  |
| **Drinking Alcohol** |  |  |
| No® |  |  |
| Yes | 1.288**** (1.124 1.477) | 0.937 (0.690 1.273) |
| **Smoking/Consuming Tobacco** |  |  |
| No® |  |  |
| Yes | 1.220**** (1.095 1.359) | 1.442**** (1.177 1.768) |
| ***Physical Activity*** |  |  |
| **Vigorous** |  |  |
| Never® |  |  |
| Rare | 1.009 (0.882 1.154) | 0.733** (0.548 0.98) |
| Everyday | 0.894 (0.79 1.011) | 0.668*** (0.530 0.842) |
| **Moderate** |  |  |
| Never® |  |  |
| Rare | 1.134 (0.966 1.331) | 0.987 (0.741 1.313) |
| Everyday | 1.023 (0.9 1.164) | 0.752** (0.601 0.941) |
| **Yoga/Pranayam** |  |  |
| Never® |  |  |
| Rare | 0.916 (0.716 1.173) | 1.986*** (1.342 2.94) |
| Everyday | 0.978 (0.816 1.171) | 1.088 (0.799 1.480) |
| **Childhood health** |  |  |
| Good/Fair® |  |  |
| Poor | 1.223 (0.904 1.655) | 1.852** (1.089 3.150) |
| ***Regions*** |  |  |
| North® |  |  |
| Central | 1.145 (0.949 1.381) | 0.672 (0.439 1.029) |
| East | 1.303*** (1.093 1.552) | 1.363 (0.941 1.975) |
| Northeast | 0.881 (0.708 1.097) | 0.908 (0.573 1.441) |
| West | 2.342**** (1.91 2.873) | 1.037 (0.723 1.488) |
| South | 2.105**** (1.759 2.519) | 1.779*** (1.269 2.494) |
| Union Territories | 1.370** (1.049 1.789) | 1.056 (0.732 1.525) |
| **Constant** | **0.579** | **2.044** |

**Note: ® reference category, ****P<0.001, ***P<0.01, **P<0.05**

**Table-S6: Result of logistic regression for FL by age-groups**

| **Covariates** | **Odds Ratio** | |
| --- | --- | --- |
|  | **60-65** | **65+** |
| **Res_CHC** | 1.667**** (1.305 2.129) | 1.340** (1.040 1.727) |
| **Type of work** |  |  |
| Formal® |  |  |
| Informal | 0.982 (0.856 1.127) | 0.797*** (0.689 0.921) |
| **Type of work*Res_CHC** | 0.911 (0.688 1.206) | 1.136 (0.847 1.525) |
| ***Socio-economic & demographic*** |  |  |
| **Gender** |  |  |
| Male® |  |  |
| Female | 2.068**** (1.773 2.412) | 1.875**** (1.579 2.227) |
| **Caste groups** |  |  |
| General® |  |  |
| Scheduled Tribe | 0.918 (0.739 1.140) | 0.691*** (0.544 0.877) |
| Scheduled Caste | 0.982 (0.807 1.195) | 0.832 (0.674 1.026) |
| Other Backward Class | 1.009 (0.854 1.192) | 0.872 (0.733 1.038) |
| **Religion** |  |  |
| Hindu® |  |  |
| Muslim | 1.239** (1.008 1.524) | 1.152 (0.929 1.429) |
| Others | 0.719*** (0.577 0.895) | 0.939 (0.756 1.168) |
| **Education level** |  |  |
| Low® |  |  |
| Middle | 0.693**** (0.576 0.833) | 0.569**** (0.467 0.694) |
| High | 0.496**** (0.355 0.694) | 0.515**** (0.370 0.716) |
| **Marital status** |  |  |
| Currently married® |  |  |
| Others | 1.132 (0.973 1.316) | 1.446**** (1.244 1.682) |
| **Place of residence** |  |  |
| Rural® |  |  |
| Urban | 0.614**** (0.523 0.721) | 0.692**** (0.587 0.816) |
| **Wealth** |  |  |
| Low® |  |  |
| Medium | 0.858** (0.746 0.987) | 0.723**** (0.618 0.845) |
| High | 0.795*** (0.670 0.945) | 0.873 (0.726 1.049) |
| **Household size** | 1.058 (0.989 1.131) | 1.031 (0.964 1.104) |
| ***Work Characteristics*** |  |  |
| **Working hours** |  |  |
| Less than 24 hours® |  |  |
| 24-48 hours | 1.115 (0.965 1.289) | 0.938 (0.81 1.088) |
| 48+ hours | 0.978 (0.834 1.148) | 0.927 (0.780 1.102) |
| **Ln(Wage)** | 0.907 (0.844 0.974) | 0.891*** (0.827 0.961) |
| **Duration being in current work** |  |  |
| Less than 15 years® |  |  |
| 15-30 years | 0.982 (0.810 1.189) | 1.100 (0.885 1.366) |
| 30-45 years | 0.946 (0.802 1.116) | 1.069 (0.876 1.305) |
| 45 years and over | 1.102 (0.918 1.323) | 1.122 (0.938 1.340) |
| ***Life style behaviour*** |  |  |
| **Drinking Alcohol** |  |  |
| No® |  |  |
| Yes | 1.141 (0.966 1.347) | 1.330*** (1.104 1.603) |
| **Smoking/Consuming Tobacco** |  |  |
| No® |  |  |
| Yes | 1.335**** (1.171 1.523) | 1.213*** (1.056 1.393) |
| ***Physical Activity*** |  |  |
| **Vigorous** |  |  |
| Never® |  |  |
| Rare | 0.990 (0.840 1.167) | 0.930 (0.779 1.109) |
| Everyday | 0.918 (0.793 1.063) | 0.789*** (0.673 0.924) |
| **Moderate** |  |  |
| Never® |  |  |
| Rare | 1.162 (0.956 1.412) | 1.028 (0.842 1.254) |
| Everyday | 1.019 (0.872 1.191) | 0.872 (0.745 1.021) |
| **Yoga/Pranayam** |  |  |
| Never® |  |  |
| Rare | 1.025 (0.783 1.342) | 1.272 (0.911 1.775) |
| Everyday | 1.061 (0.854 1.320) | 0.888 (0.712 1.108) |
| **Childhood health** |  |  |
| Good/Fair® |  |  |
| Poor | 1.461** (1.03 2.073) | 1.196 (0.807 1.771) |
| ***Regions*** |  |  |
| North® |  |  |
| Central | 1.085 (0.864 1.363) | 0.967 (0.748 1.250) |
| East | 1.280** (1.034 1.584) | 1.269** (1.004 1.605) |
| Northeast | 0.947 (0.722 1.243) | 0.82 (0.615 1.094) |
| West | 1.995**** (1.570 2.535) | 1.718**** (1.331 2.217) |
| South | 1.914**** (1.539 2.379) | 2.042**** (1.621 2.573) |
| Union Territories | 1.168 (0.869 1.569) | 1.369** (1.016 1.845) |
| **Constant** | **0.621** | **1.908** |

**Note: ® reference category, ****P<0.001, ***P<0.01, **P<0.05**

**Poor Cognitive Functioning**

**Table-S7: Result of logistic regression for PCF by gender**

| **Covariates** | **Odds Ratio** | |
| --- | --- | --- |
|  | **Male** | **Female** |
| **Res_CHC** | 1.008 (0.749 1.358) | 1.004 (0.698 1.445) |
| **Type of work** |  |  |
| Formal® |  |  |
| Informal | 1.207** (1.027 1.419) | 1.100 (0.906 1.336) |
| **Type of work*Res_CHC** | 0.936 (0.668 1.311) | 0.889 (0.594 1.328) |
| ***Socio-economic & demographic*** |  |  |
| **Age groups** |  |  |
| 60-65® |  |  |
| 65+ | 1.428**** (1.239 1.645) | 1.279*** (1.088 1.503) |
| **Caste groups** |  |  |
| General® |  |  |
| Scheduled Tribe | 1.905**** (1.493 2.431) | 1.712**** (1.278 2.293) |
| Scheduled Caste | 1.215 (0.963 1.534) | 1.044 (0.803 1.358) |
| Other Backward Class | 0.895 (0.728 1.100) | 1.020 (0.804 1.294) |
| **Religion** |  |  |
| Hindu® |  |  |
| Muslim | 0.994 (0.787 1.255) | 1.078 (0.778 1.494) |
| Others | 0.984 (0.776 1.248) | 1.060 (0.806 1.394) |
| **Education level** |  |  |
| Low® |  |  |
| Middle | 0.113**** (0.080 0.160) | 0.112**** (0.051 0.246) |
| High | 0.099**** (0.046 0.211) | 1.000 |
| **Marital status** |  |  |
| Currently married® |  |  |
| Others | 1.412**** (1.175 1.696) | 1.476**** (1.260 1.729) |
| **Place of residence** |  |  |
| Rural® |  |  |
| Urban | 0.431**** (0.351 0.529) | 0.459**** (0.371 0.569) |
| **Wealth** |  |  |
| Low® |  |  |
| Medium | 0.530**** (0.453 0.62) | 0.564**** (0.470 0.678) |
| High | 0.412**** (0.336 0.505) | 0.406**** (0.320 0.515) |
| **Household size** | 1.090** (1.007 1.179) | 1.106** (1.025 1.194) |
| ***Work Characteristics*** |  |  |
| **Working hours** |  |  |
| Less than 24 hours® |  |  |
| 24-48 hours | 0.851 (0.722 1.002) | 0.763*** (0.639 0.912) |
| 48+ hours | 0.950 (0.792 1.141) | 0.720*** (0.581 0.893) |
| **Ln(Wage)** | 0.787**** (0.724 0.856) | 0.886** (0.808 0.972) |
| **Duration being in current work** |  |  |
| Less than 15 years® |  |  |
| 15-30 years | 1.123 (0.888 1.420) | 1.003 (0.775 1.300) |
| 30-45 years | 0.965 (0.783 1.188) | 1.184 (0.945 1.483) |
| 45 years and over | 1.101 (0.901 1.344) | 1.548**** (1.239 1.934) |
| ***Life style behaviour*** |  |  |
| **Drinking Alcohol** |  |  |
| No® |  |  |
| Yes | 1.331*** (1.132 1.566) | 2.137**** (1.481 3.084) |
| **Smoking/Consuming Tobacco** |  |  |
| No® |  |  |
| Yes | 1.003 (0.864 1.165) | 0.969 (0.816 1.151) |
| ***Physical Activity*** |  |  |
| **Vigorous** |  |  |
| Never® |  |  |
| Rare | 1.039 (0.859 1.256) | 0.981 (0.793 1.214) |
| Everyday | 0.922 (0.776 1.095) | 0.923 (0.760 1.122) |
| **Moderate** |  |  |
| Never® |  |  |
| Rare | 0.669**** (0.544 0.823) | 0.488**** (0.364 0.653) |
| Everyday | 0.664**** (0.561 0.785) | 0.534**** (0.427 0.667) |
| **Yoga/Pranayam** |  |  |
| Never® |  |  |
| Rare | 0.693 (0.479 1.003) | 1.137 (0.746 1.732) |
| Everyday | 0.649*** (0.492 0.856) | 0.887 (0.644 1.221) |
| **Childhood health** |  |  |
| Good/Fair® |  |  |
| Poor | 0.552** (0.341 0.893) | 0.694 (0.377 1.278) |
| ***Regions*** |  |  |
| North® |  |  |
| Central | 0.911 (0.696 1.191) | 0.803 (0.585 1.103) |
| East | 1.037 (0.81 1.328) | 1.083 (0.800 1.467) |
| Northeast | 0.882 (0.648 1.2) | 0.563*** (0.391 0.810) |
| West | 1.266 (0.942 1.701) | 1.469** (1.082 1.994) |
| South | 1.414*** (1.096 1.823) | 0.947 (0.709 1.267) |
| Union Territories | 1.076 (0.76 1.524) | 0.778 (0.531 1.138) |
| **Constant** | **2.367** | **3.239** |

**Note: ® reference category, ****P<0.001, ***P<0.01, **P<0.05**

**Table-S8: Result of logistic regression for PCF by place of residence**

| **Covariates** | **Odds Ratio** | |
| --- | --- | --- |
|  | **Rural** | **Urban** |
| **Res_CHC** | 0.974 (0.756 1.255) | 1.242 (0.713 2.164) |
| **Type of work** |  |  |
| Formal® |  |  |
| Informal | 1.145** (0.999 1.312) | 1.245 (0.916 1.691) |
| **Type of work*Res_CHC** | 0.942 (0.709 1.250) | 0.765 (0.409 1.433) |
| ***Socio-economic & demographic*** |  |  |
| **Gender** |  |  |
| Male® |  |  |
| Female | 2.559**** (2.230 2.938) | 4.058**** (2.867 5.742) |
| **Age groups** |  |  |
| 60-65® |  |  |
| 65+ | 1.266**** (1.127 1.422) | 1.945**** (1.492 2.534) |
| **Caste groups** |  |  |
| General® |  |  |
| Scheduled Tribe | 1.807**** (1.472 2.218) | 1.864** (1.134 3.063) |
| Scheduled Caste | 1.093 (0.901 1.327) | 1.186 (0.794 1.773) |
| Other Backward Class | 0.908 (0.762 1.081) | 1.051 (0.738 1.496) |
| **Religion** |  |  |
| Hindu® |  |  |
| Muslim | 1.064 (0.854 1.326) | 0.840 (0.579 1.217) |
| Others | 0.906 (0.747 1.098) | 1.680** (1.051 2.686) |
| **Education level** |  |  |
| Low® |  |  |
| Middle | 0.097**** (0.066 0.143) | 0.146**** (0.083 0.257) |
| High | 0.077**** (0.028 0.210) | 0.083**** (0.026 0.267) |
| **Marital status** |  |  |
| Currently married® |  |  |
| Others | 1.432**** (1.258 1.630) | 1.390**** (1.031 1.874) |
| **Wealth** |  |  |
| Low® |  |  |
| Medium | 0.526**** (0.461 0.599) | 0.638*** (0.472 0.863) |
| High | 0.406**** (0.344 0.479) | 0.422**** (0.274 0.648) |
| **Household size** | 1.105*** (1.041 1.174) | 1.117 (0.978 1.276) |
| ***Work Characteristics*** |  |  |
| **Working hours** |  |  |
| Less than 24 hours® |  |  |
| 24-48 hours | 0.782**** (0.686 0.891) | 0.945 (0.697 1.281) |
| 48+ hours | 0.844** (0.725 0.982) | 0.725 (0.51 1.03) |
| **Ln(Wage)** | 0.811**** (0.757 0.868) | 0.877 (0.757 1.017) |
| **Duration being in current work** |  |  |
| Less than 15 years® |  |  |
| 15-30 years | 0.998 (0.815 1.222) | 1.247 (0.876 1.776) |
| 30-45 years | 1.058 (0.892 1.256) | 1.09 (0.763 1.557) |
| 45 years and over | 1.344**** (1.139 1.586) | 0.945 (0.649 1.375) |
| ***Life style behaviour*** |  |  |
| **Drinking Alcohol** |  |  |
| No® |  |  |
| Yes | 1.521**** (1.304 1.774) | 1.372 (0.901 2.091) |
| **Smoking/Consuming Tobacco** |  |  |
| No® |  |  |
| Yes | 0.886** (0.784 1.001) | 1.539*** (1.154 2.051) |
| ***Physical Activity*** |  |  |
| **Vigorous** |  |  |
| Never® |  |  |
| Rare | 0.949 (0.815 1.105) | 1.317 (0.887 1.956) |
| Everyday | 0.855** (0.742 0.984) | 1.217 (0.882 1.680) |
| **Moderate** |  |  |
| Never® |  |  |
| Rare | 0.639**** (0.532 0.769) | 0.548*** (0.361 0.831) |
| Everyday | 0.693**** (0.599 0.802) | 0.437**** (0.317 0.602) |
| **Yoga/Pranayam** |  |  |
| Never® |  |  |
| Rare | 0.945 (0.704 1.268) | 0.537 (0.262 1.101) |
| Everyday | 0.766** (0.613 0.957) | 0.515** (0.291 0.911) |
| **Childhood health** |  |  |
| Good/Fair® |  |  |
| Poor | 0.631** (0.421 0.944) | 0.471 (0.172 1.289) |
| ***Regions*** |  |  |
| North® |  |  |
| Central | 0.927 (0.746 1.153) | 0.588 (0.327 1.057) |
| East | 1.148 (0.937 1.407) | 0.719 (0.421 1.226) |
| Northeast | 0.878 (0.683 1.129) | 0.226**** (0.113 0.451) |
| West | 1.503*** (1.194 1.893) | 0.897 (0.545 1.478) |
| South | 1.330*** (1.080 1.637) | 0.702 (0.438 1.126) |
| Union Territories | 0.936 (0.69 1.269) | 0.741 (0.444 1.237) |
| **Constant** | **2.073** | **0.357** |

**Note: ® reference category, ****P<0.001, ***P<0.01, **P<0.05**

**Table-S9: Result of logistic regression for PCF by age-group**

| **Covariates** | **Odds Ratio** | |
| --- | --- | --- |
|  | **60-65** | **65+** |
| **Res_CHC** | 0.873 (0.630 1.209) | 1.143 (0.827 1.581) |
| **Type of work** |  |  |
| Formal® |  |  |
| Informal | 1.135 (0.954 1.351) | 1.196** (1.003 1.427) |
| **Type of work*Res_CHC** | 1.134 (0.788 1.63) | 0.743 (0.516 1.07) |
| ***Socio-economic & demographic*** |  |  |
| **Gender** |  |  |
| Male® |  |  |
| Female | 2.853**** (2.393 3.402) | 2.599**** (2.155 3.135) |
| **Caste groups** |  |  |
| General® |  |  |
| Scheduled Tribe | 2.001**** (1.547 2.588) | 1.687**** (1.284 2.216) |
| Scheduled Caste | 1.238 (0.969 1.58) | 1.029 (0.803 1.318) |
| Other Backward Class | 1.011 (0.81 1.263) | 0.878 (0.706 1.09) |
| **Religion** |  |  |
| Hindu® |  |  |
| Muslim | 1.004 (0.768 1.311) | 1.046 (0.806 1.358) |
| Others | 0.839 (0.653 1.079) | 1.181 (0.916 1.522) |
| **Education level** |  |  |
| Low® |  |  |
| Middle | 0.100**** (0.062 0.161) | 0.120**** (0.078 0.184) |
| High | 0.025**** (0.004 0.182) | 0.124**** (0.054 0.285) |
| **Marital status** |  |  |
| Currently married® |  |  |
| Others | 1.234** (1.043 1.46) | 1.623**** (1.373 1.919) |
| **Place of residence** |  |  |
| Rural® |  |  |
| Urban | 0.376**** (0.304 0.467) | 0.522**** (0.426 0.64) |
| **Wealth** |  |  |
| Low® |  |  |
| Medium | 0.514**** (0.438 0.604) | 0.583**** (0.488 0.697) |
| High | 0.360**** (0.29 0.446) | 0.469**** (0.376 0.586) |
| **Household size** | 1.136*** (1.052 1.228) | 1.061 (0.983 1.146) |
| ***Work Characteristics*** |  |  |
| **Working hours** |  |  |
| Less than 24 hours® |  |  |
| 24-48 hours | 0.817** (0.689 0.968) | 0.793*** (0.669 0.939) |
| 48+ hours | 0.900 (0.744 1.089) | 0.762*** (0.622 0.934) |
| **Ln(Wage)** | 0.802**** (0.735 0.875) | 0.847**** (0.776 0.925) |
| **Duration being in current work** |  |  |
| Less than 15 years® |  |  |
| 15-30 years | 1.020 (0.806 1.291) | 1.136 (0.873 1.478) |
| 30-45 years | 1.048 (0.857 1.28) | 1.002 (0.786 1.278) |
| 45 years and over | 1.183 (0.955 1.466) | 1.376*** (1.117 1.696) |
| ***Life style behaviour*** |  |  |
| **Drinking Alcohol** |  |  |
| No® |  |  |
| Yes | 1.472**** (1.209 1.791) | 1.539**** (1.243 1.905) |
| **Smoking/Consuming Tobacco** |  |  |
| No® |  |  |
| Yes | 0.942 (0.806 1.101) | 1.01 (0.86 1.186) |
| ***Physical Activity*** |  |  |
| **Vigorous** |  |  |
| Never® |  |  |
| Rare | 0.947 (0.779 1.152) | 1.063 (0.866 1.304) |
| Everyday | 0.874 (0.733 1.044) | 0.962 (0.798 1.158) |
| **Moderate** |  |  |
| Never® |  |  |
| Rare | 0.626**** (0.493 0.795) | 0.617**** (0.487 0.783) |
| Everyday | 0.670**** (0.555 0.808) | 0.601**** (0.499 0.723) |
| **Yoga/Pranayam** |  |  |
| Never® |  |  |
| Rare | 0.899 (0.636 1.27) | 0.825 (0.534 1.274) |
| Everyday | 0.600**** (0.441 0.817) | 0.833 (0.629 1.103) |
| **Childhood health** |  |  |
| Good/Fair® |  |  |
| Poor | 0.596 (0.352 1.011) | 0.62 (0.367 1.05) |
| ***Regions*** |  |  |
| North® |  |  |
| Central | 0.786 (0.597 1.033) | 0.947 (0.7 1.282) |
| East | 1.039 (0.804 1.343) | 1.074 (0.811 1.422) |
| Northeast | 0.775** (0.563 1.068) | 0.688** (0.488 0.97) |
| West | 1.4 (1.05 1.867) | 1.363** (1.008 1.843) |
| South | 1.061 (0.815 1.381) | 1.296 (0.984 1.706) |
| Union Territories | 0.82 (0.57 1.18) | 1.039 (0.723 1.494) |
| **Constant** | **2.230** | **1.780** |

**Note: ® reference category, ****P<0.001, ***P<0.01, **P<0.05**

**Poor Cognitive functioning and/or Functional Limitation**

**Table-S10: Result of logistic regression for PCF and/or FL by gender**

| **Covariates** | **Odds Ratio** | |
| --- | --- | --- |
|  | **Male** | **Female** |
| **Res_CHC** | 1.334*** (1.088 1.637) | 1.429 (0.975 2.094) |
| **Type of work** |  |  |
| Formal® |  |  |
| Informal | 1.065 (0.948 1.196) | 0.960 (0.781 1.180) |
| **Type of work*Res_CHC** | 1.027 (0.809 1.304) | 0.814 (0.532 1.245) |
| ***Socio-economic & demographic*** |  |  |
| **Age groups** |  |  |
| 60-65® |  |  |
| 65+ | 1.393**** (1.251 1.551) | 1.556**** (1.302 1.860) |
| **Caste groups** |  |  |
| General® |  |  |
| Scheduled Tribe | 1.200 (0.991 1.453) | 1.184 (0.868 1.616) |
| Scheduled Caste | 0.950 (0.800 1.127) | 0.891 (0.675 1.175) |
| Other Backward Class | 0.887 (0.771 1.020) | 0.961 (0.748 1.234) |
| **Religion** |  |  |
| Hindu® |  |  |
| Muslim | 1.188** (1.005 1.404) | 0.947 (0.663 1.352) |
| Others | 0.875 (0.727 1.054) | 0.825 (0.623 1.093) |
| **Education level** |  |  |
| Low® |  |  |
| Middle | 0.441**** (0.383 0.507) | 0.392**** (0.262 0.587) |
| High | 0.381**** (0.297 0.488) | 0.394*** (0.201 0.773) |
| **Marital status** |  |  |
| Currently married® |  |  |
| Others | 1.418**** (1.219 1.649) | 1.381**** (1.164 1.638) |
| **Place of residence** |  |  |
| Rural® |  |  |
| Urban | 0.565**** (0.493 0.647) | 0.490**** (0.396 0.606) |
| **Wealth** |  |  |
| Low® |  |  |
| Medium | 0.628**** (0.555 0.712) | 0.661**** (0.542 0.806) |
| High | 0.598**** (0.516 0.693) | 0.548**** (0.429 0.700) |
| **Household size** | 1.066** (1.002 1.134) | 1.085 (0.999 1.179) |
| ***Work Characteristics*** |  |  |
| **Working hours** |  |  |
| Less than 24 hours® |  |  |
| 24-48 hours | 0.930 (0.820 1.055) | 0.856 (0.706 1.038) |
| 48+ hours | 0.956 (0.832 1.098) | 0.772** (0.613 0.972) |
| **Ln(Wage)** | 0.842**** (0.790 0.896) | 0.915 (0.830 1.009) |
| **Duration being in current work** |  |  |
| Less than 15 years® |  |  |
| 15-30 years | 1.044 (0.879 1.239) | 0.846 (0.651 1.100) |
| 30-45 years | 0.889 (0.765 1.035) | 0.963 (0.761 1.219) |
| 45 years and over | 1.109 (0.952 1.292) | 1.169 (0.916 1.491) |
| ***Life style behaviour*** |  |  |
| **Drinking Alcohol** |  |  |
| No® |  |  |
| Yes | 1.351**** (1.184 1.541) | 2.476**** (1.522 4.028) |
| **Smoking/Consuming Tobacco** |  |  |
| No® |  |  |
| Yes | 1.143** (1.022 1.279) | 1.052 (0.872 1.271) |
| ***Physical Activity*** |  |  |
| **Vigorous** |  |  |
| Never® |  |  |
| Rare | 1.035 (0.894 1.197) | 0.938 (0.747 1.178) |
| Everyday | 0.847** (0.744 0.963) | 0.937 (0.762 1.153) |
| **Moderate** |  |  |
| Never® |  |  |
| Rare | 0.962 (0.821 1.127) | 0.739 (0.537 1.015) |
| Everyday | 0.836*** (0.735 0.950) | 0.695*** (0.544 0.888) |
| **Yoga/Pranayam** |  |  |
| Never® |  |  |
| Rare | 0.955 (0.748 1.219) | 1.236 (0.804 1.902) |
| Everyday | 0.940 (0.788 1.120) | 1.003 (0.723 1.391) |
| **Childhood health** |  |  |
| Good/Fair® |  |  |
| Poor | 1.062 (0.785 1.436) | 1.319 (0.683 2.550) |
| ***Regions*** |  |  |
| North® |  |  |
| Central | 0.966 (0.792 1.177) | 1.039 (0.749 1.442) |
| East | 1.250** (1.042 1.499) | 1.743*** (1.259 2.413) |
| Northeast | 0.953 (0.758 1.198) | 0.809 (0.563 1.162) |
| West | 1.463*** (1.179 1.816) | 1.901**** (1.371 2.637) |
| South | 1.813**** (1.503 2.189) | 1.741**** (1.284 2.360) |
| Union Territories | 1.106 (0.857 1.427) | 0.986 (0.675 1.441) |
| **Constant** | **3.384** | **5.741** |

**Note: ® reference category, ****P<0.001, ***P<0.01, **P<0.05**

**Table-S11: Result of logistic regression for PCF and/or FL by place of residence**

| **Covariates** | **Odds Ratio** | |
| --- | --- | --- |
|  | **Rural** | **Urban** |
| **Res_CHC** | 1.309** (1.058 1.620) | 1.449** (1.035 2.029) |
| **Type of work** |  |  |
| Formal® |  |  |
| Informal | 1.068 (0.949 1.202) | 0.977 (0.801 1.192) |
| **Type of work*Res_CHC** | 0.939 (0.736 1.198) | 1.141 (0.762 1.708) |
| ***Socio-economic & demographic*** |  |  |
| **Gender** |  |  |
| Male® |  |  |
| Female | 2.668**** (2.334 3.049) | 2.911**** (2.261 3.749) |
| **Age groups** |  |  |
| 60-65® |  |  |
| 65+ | 1.345**** (1.209 1.496) | 1.767**** (1.468 2.126) |
| **Caste groups** |  |  |
| General® |  |  |
| Scheduled Tribe | 1.176 (0.976 1.418) | 0.996 (0.682 1.455) |
| Scheduled Caste | 0.911 (0.768 1.080) | 0.965 (0.719 1.295) |
| Other Backward Class | 0.878 (0.758 1.018) | 0.978 (0.775 1.233) |
| **Religion** |  |  |
| Hindu® |  |  |
| Muslim | 1.092 (0.901 1.324) | 1.248 (0.971 1.604) |
| Others | 0.826** (0.693 0.984) | 1.003 (0.721 1.396) |
| **Education level** |  |  |
| Low® |  |  |
| Middle | 0.434**** (0.370 0.510) | 0.418**** (0.329 0.532) |
| High | 0.322**** (0.229 0.452) | 0.402**** (0.287 0.564) |
| **Marital status** |  |  |
| Currently married® |  |  |
| Others | 1.454**** (1.277 1.655) | 1.257** (1.001 1.578) |
| **Wealth** |  |  |
| Low® |  |  |
| Medium | 0.566**** (0.500 0.640) | 0.847 (0.683 1.049) |
| High | 0.494**** (0.426 0.572) | 0.927 (0.715 1.202) |
| **Household size** | 1.106*** (1.045 1.171) | 1.012 (0.917 1.117) |
| ***Work Characteristics*** |  |  |
| **Working hours** |  |  |
| Less than 24 hours® |  |  |
| 24-48 hours | 0.916 (0.812 1.033) | 0.873 (0.699 1.090) |
| 48+ hours | 0.911 (0.794 1.046) | 0.829 (0.652 1.054) |
| **Ln(Wage)** | 0.875**** (0.822 0.931) | 0.810**** (0.730 0.900) |
| **Duration being in current work** |  |  |
| Less than 15 years® |  |  |
| 15-30 years | 0.962 (0.805 1.149) | 1.029 (0.803 1.320) |
| 30-45 years | 0.924 (0.795 1.074) | 0.912 (0.715 1.164) |
| 45 years and over | 1.145 (0.986 1.331) | 1.040 (0.788 1.371) |
| ***Life style behaviour*** |  |  |
| **Drinking Alcohol** |  |  |
| No® |  |  |
| Yes | 1.497**** (1.302 1.722) | 1.180 (0.884 1.575) |
| **Smoking/Consuming Tobacco** |  |  |
| No® |  |  |
| Yes | 1.032 (0.924 1.153) | 1.409*** (1.155 1.719) |
| ***Physical Activity*** |  |  |
| **Vigorous** |  |  |
| Never® |  |  |
| Rare | 1.004 (0.874 1.154) | 0.940 (0.711 1.243) |
| Everyday | 0.857** (0.754 0.973) | 0.779** (0.623 0.973) |
| **Moderate** |  |  |
| Never® |  |  |
| Rare | 0.935 (0.793 1.101) | 0.934 (0.705 1.237) |
| Everyday | 0.883 (0.773 1.008) | 0.680*** (0.546 0.848) |
| **Yoga/Pranayam** |  |  |
| Never® |  |  |
| Rare | 0.856 (0.67 1.095) | 1.697*** (1.144 2.516) |
| Everyday | 0.937 (0.782 1.123) | 1.006 (0.743 1.360) |
| **Childhood health** |  |  |
| Good/Fair® |  |  |
| Poor | 0.951 (0.695 1.303) | 1.618 (0.944 2.775) |
| ***Regions*** |  |  |
| North® |  |  |
| Central | 1.056 (0.875 1.274) | 0.650** (0.432 0.977) |
| East | 1.366*** (1.145 1.629) | 1.353 (0.941 1.944) |
| Northeast | 0.95 (0.765 1.179) | 0.709 (0.454 1.106) |
| West | 1.912**** (1.543 2.369) | 1.04 (0.734 1.474) |
| South | 1.912**** (1.591 2.298) | 1.540** (1.108 2.142) |
| Union Territories | 1.067 (0.808 1.408) | 0.956 (0.671 1.362) |
| **Constant** | **2.425** | **2.922** |

**Note: ® reference category, ****P<0.001, ***P<0.01, **P<0.05**

**Table-S12: Result of logistic regression for PCF and/or FL by age groups**

| **Covariates** | **Odds Ratio** | |
| --- | --- | --- |
|  | **60-65** | **65+** |
| **Res_CHC** | 1.452*** (1.136 1.857) | 1.202 (0.924 1.564) |
| **Type of work** |  |  |
| Formal® |  |  |
| Informal | 1.098 (0.957 1.259) | 0.951 (0.819 1.105) |
| **Type of work*Res_CHC** | 0.940 (0.709 1.246) | 1.031 (0.760 1.400) |
| ***Socio-economic & demographic*** |  |  |
| **Gender** |  |  |
| Male® |  |  |
| Female | 2.787**** (2.390 3.250) | 2.745**** (2.279 3.305) |
| **Caste groups** |  |  |
| General® |  |  |
| Scheduled Tribe | 1.350*** (1.087 1.676) | 1.055 (0.824 1.351) |
| Scheduled Caste | 1.065 (0.876 1.295) | 0.787** (0.633 0.979) |
| Other Backward Class | 0.974 (0.824 1.151) | 0.829** (0.692 0.992) |
| **Religion** |  |  |
| Hindu® |  |  |
| Muslim | 1.164 (0.947 1.430) | 1.134 (0.907 1.418) |
| Others | 0.731*** (0.591 0.905) | 1.016 (0.811 1.272) |
| **Education level** |  |  |
| Low® |  |  |
| Middle | 0.459**** (0.383 0.550) | 0.403**** (0.331 0.489) |
| High | 0.367**** (0.263 0.513) | 0.394**** (0.284 0.548) |
| **Marital status** |  |  |
| Currently married® |  |  |
| Others | 1.165 (0.998 1.361) | 1.637**** (1.392 1.924) |
| **Place of residence** |  |  |
| Rural® |  |  |
| Urban | 0.484**** (0.413 0.567) | 0.617**** (0.522 0.730) |
| **Wealth** |  |  |
| Low® |  |  |
| Medium | 0.639**** (0.556 0.735) | 0.622**** (0.529 0.731) |
| High | 0.540**** (0.456 0.641) | 0.638**** (0.527 0.771) |
| **Household size** | 1.090** (1.018 1.166) | 1.048 (0.975 1.126) |
| ***Work Characteristics*** |  |  |
| **Working hours** |  |  |
| Less than 24 hours® |  |  |
| 24-48 hours | 0.986 (0.853 1.139) | 0.831** (0.712 0.970) |
| 48+ hours | 0.983 (0.838 1.152) | 0.813** (0.679 0.972) |
| **Ln(Wage)** | 0.866**** (0.806 0.930) | 0.857**** (0.792 0.927) |
| **Duration being in current work** |  |  |
| Less than 15 years® |  |  |
| 15-30 years | 0.955 (0.789 1.154) | 1.022 (0.817 1.279) |
| 30-45 years | 0.896 (0.76 1.055) | 0.907 (0.739 1.114) |
| 45 years and over | 1.14 (0.949 1.371) | 1.150 (0.956 1.384) |
| ***Life style behaviour*** |  |  |
| **Drinking Alcohol** |  |  |
| No® |  |  |
| Yes | 1.361**** (1.155 1.602) | 1.553**** (1.278 1.887) |
| **Smoking/Consuming Tobacco** |  |  |
| No® |  |  |
| Yes | 1.120 (0.983 1.276) | 1.096 (0.951 1.264) |
| ***Physical Activity*** |  |  |
| **Vigorous** |  |  |
| Never® |  |  |
| Rare | 0.953 (0.808 1.125) | 1.063 (0.884 1.279) |
| Everyday | 0.850** (0.734 0.984) | 0.873 (0.741 1.028) |
| **Moderate** |  |  |
| Never® |  |  |
| Rare | 1.004 (0.826 1.220) | 0.839 (0.682 1.031) |
| Everyday | 0.875 (0.750 1.022) | 0.749*** (0.635 0.882) |
| **Yoga/Pranayam** |  |  |
| Never® |  |  |
| Rare | 0.913 (0.699 1.193) | 1.292 (0.914 1.825) |
| Everyday | 0.957 (0.771 1.188) | 0.921 (0.737 1.150) |
| **Childhood health** |  |  |
| Good/Fair® |  |  |
| Poor | 1.196 (0.831 1.721) | 0.967 (0.645 1.449) |
| ***Regions*** |  |  |
| North® |  |  |
| Central | 0.985 (0.788 1.231) | 0.933 (0.719 1.210) |
| East | 1.306** (1.059 1.611) | 1.328** (1.045 1.688) |
| Northeast | 0.899 (0.692 1.167) | 0.87 (0.652 1.161) |
| West | 1.780**** (1.397 2.268) | 1.345** (1.032 1.753) |
| South | 1.614**** (1.300 2.004) | 1.915**** (1.508 2.431) |
| Union Territories | 0.940 (0.703 1.257) | 1.175 (0.860 1.605) |
| **Constant** | **2.361** | **5.344** |

**Note: ® reference category, ****P<0.001, ***P<0.01, **P<0.05**

**Table-S13: Work classification**

| **Type of work** | **Description** |
| --- | --- |
| Formal workers | Legislators, senior officials and Managers |
|  | Professionals |
|  | Technicians and associate professionals |
|  | Clerks |
|  | Service workers and shop and market sales workers |
|  | Market oriented skilled agricultural and fishery workers |
| Informal workers | Subsistence agricultural and fishery workers |
|  | Craft and related trade workers |
|  | Plant and machine operators and assemblers |
|  | Elementary occupations (e.g., agricultural labour, construction labour etc.) |
|  | Other unclassified workers (e.g., housemaid, gardener etc.) |

**Table-S14: Indicators under functional limitations**

| **Difficulties in performing few everyday activities** | **Response** |
| --- | --- |
| Dressing, including putting on chappals, shoes, etc. | 1. Yes, 2. No |
| Walking across a room | 1. Yes, 2. No |
| Bathing | 1. Yes, 2. No |
| Eating, difficulties | 1. Yes, 2. No |
| Getting in or out of bed | 1. Yes, 2. No |
| Using the toilet, including getting up and down | 1. Yes, 2. No |
| Preparing a hot meal (cooking and serving) | 1. Yes, 2. No |
| Shopping for groceries | 1. Yes, 2. No |
| Making telephone calls | 1. Yes, 2. No |
| Taking medications | 1. Yes, 2. No |
| Doing work around the house or garden | 1. Yes, 2. No |
| Managing money, such as paying bills and keeping track of expenses | 1. Yes, 2. No |
| Getting around or finding address in unfamiliar place | 1. Yes, 2. No |
