# Supplementary material for: Informal sector employment and the health outcomes of older workers in India
Source: PLoS One. 2023 Feb 22;18(2):e0266576. doi: 10.1371/journal.pone.0266576 (PMC9946227; doi:10.1371/journal.pone.0266576)
Supplement: Attachment — Submitted filename: PLoS report_20220530.docx [file pone.0266576.s002.docx]

This paper uses the baseline wave of longitudinal Ageing Study in India (LASI) to investigate the association between health and employment in later years, as well as the heterogeneous effect by employment type. The analytical sample contains only working populations over age 60. Overall, the paper is well written in a clear and scientific matter. Empirical results consistently show the health effect varies by employment type and specific health outcomes. Employment type significantly moderates the effect of CHC on FL, and FL on PCF.

**Comments:**

My major comments are on the complexity of employment in later years as established by existing research (e.g., Rietveld et al.,2014; van Zon et al.,2020). It is understandable that the cross-sectional nature of the dataset limits further exploration of any dynamic aspects of employment, also, the goal of the paper is not aimed at a causal identification. However, it could be more informative to add some relevant descriptive statistics, sensitivity checks, or comments regarding the defined employment status that might not truly reflect the general associations.

1) The observed employment type might contain some noise due to potential complex work transitions in later years such as partial retirement. Some transitions and jobs could be quite recent and temporary, especially in the informal sector, which may require additional control for working characteristics such as working years and sectors (public/private).

Without any further details, it is possible that some people are working as a recovery from an early retirement or mandatory retirement, or at a phase of bridge job. For one thing, the current employment can differ much from their main career which determinants one’s pension and other welfare schemes.

For another, the health effect of employment might be biased given potentially complex sorting into employment for older workers. For instance, if one transited from a full-time office-based job to self-employment to better manage working hours, the estimated health effect of the informal sector might vary by time. Socioeconomically advantaged individuals might extend working life as a way to continue social participation which probably exerts a positive impact on mental health and well-being, in the meanwhile, affecting physical health.

2) It would be better to add separate estimation by gender considering the gender difference in terms of both health, healthcare utilisation and employment aspects (e.g., Kandrack et al.,1991). Especially, labour force participation is more selective among females than males, and there are also great heterogeneities across occupations within formal or informal sectors. For instance, continued employment may buffer against risk factors that aggravate women’s cognitive health (Oi, Katsuya 2019).

For other minor comments:

3) It might be better to cluster standard error at a household level to account for the potential correlation among employment/retirement decisions within a household. Old couples might have a joint retirement (or working) plan.
4) The implication about the healthcare services needs could probably be better supported by adding some descriptive statistics about healthcare utilisation, and healthcare entitlements (if data permits).
5) Potential measurement error due to cognitive impairment. To what extent the estimated might be affected by the recall error in covariates such as wealth and job characteristics? Any potential screening criteria for cognitive impairment? Are surveyed people all self-interviewed or include proxy answers?

**Typos:**

P17: informal workers without CHC have 0.8 *folds* less odds

P26: those determinants which *have* considerable impacts

**Reference:**

1) Kandrack, Mary-Anne, Karen R. Grant, and Alexander Segall. "Gender differences in health related behaviour: some unanswered questions." *Social science & medicine* 32.5 (1991): 579-590.
2) Oi, Katsuya. "Does gender differentiate the effects of retirement on cognitive health?." *Research on Aging* 41.6 (2019): 575-601.
3) Rietveld, Cornelius A., Hans van Kippersluis, and A. Roy Thurik. "Self‐employment and health: Barriers or benefits?." *Health economics* 24.10 (2015): 1302-1313.
4) van Zon, Sander KR, et al. "Multimorbidity and the transition out of full-time paid employment: a longitudinal analysis of the health and retirement study." *The Journals of Gerontology: Series B* 75.3 (2020): 705-715.
