# Supplementary material for: Informal sector employment and the health outcomes of older workers in India
Source: PLoS One. 2023 Feb 22;18(2):e0266576. doi: 10.1371/journal.pone.0266576 (PMC9946227; doi:10.1371/journal.pone.0266576)
Supplement: Attachment — Submitted filename: Response_to_Reviewer.docx [file pone.0266576.s003.docx]

We would like to thank the reviewers for their useful comments. Please consider our responses below. Please note that all references to line numbers in this response letter relate to line numbers in the document with track changes.

**Reviewer reports:**

***Reviewer 1:***

Major Points to be addressed:

**Reviewer 1 - Comment#1:** As a general comment, I think the authors should address the role of selection in their analysis: those who work over the retirement age are usually either at the top or bottom of the income distribution for different reasons. In particular, those who are at the bottom of the income distribution indeed are those who need to work to make their ends meet. In this context, if the sample includes primarily individuals working in the informal/formal sector belonging to the bottom of the income distribution, I suspect the role of selection: since health status is correlated with socioeconomic conditions, you are looking at people with lower health capital and attributing to them an effect of working longer on their health status, which is instead due to an initial lower health endowment. Thus, you are looking at selected individuals who are likely to have poor health status in general.

Another important question which has not been addressed is the reverse association (given that we cannot claim causality) between the type of work and health conditions. What if individuals with cognitive deficits, limitations in functioning (and chronic diseases) are more likely to find a job in the informal (or formal) sector and work after age 60 (to make their ends meet)? Thus, if this is the case, then it could be that having pre-existing health conditions predicts the chance of working in certain types of jobs, which is associated with higher chances of having these health conditions lately.

Overall, I think that the paper should clarify from the beginning that this study looks at the association between formal/informal sectors and health outcomes, and it is not claiming causality.

**Reply:** We thank the reviewer for this useful comment.

We agree with the reviewer on the comments regarding the potential issues of selection and reverse-causation. We believe that addressing these issues will improve the overall presentation of this manuscript. First, we acknowledge the reviewer’s suggestion to clarify from the beginning that this study looks at the association between formal/informal sectors and health outcomes (page 5, lines 12-19). Based on reviewer’s comments, we also have done some modifications in our analysis (page 10, lines 12, 22-24; page 11, lines 21-25).

Studies elsewhere found that prolonged working beyond retirement age influences health conditions of the older population. In that context little has been explored on working life and its influence on the health outcomes of Indians in older age. In this study we analysed how type of formal/informal sector employment among older people (60 years and more) influences their health outcomes. While in India older population suffers from poor health conditions irrespective of their socio-economic status, few studies have focused on the sector of employment in this age group and how it influences their health. We agree with the reviewer’s concern on individuals self-selecting themselves into the formal/informal nature of employment based on their pre-existing health conditions. Meaning that there may be two-way relationship where the pre-existing health conditions influencing the type of employment and vice versa. However, this research is about how the prolonged working of older people beyond their retirement age in the formal/informal sector of employment influences their health outcomes. We believe that it is unlikely for individuals to self-selecting into the type of employment due to their pre-existing health status. We agree with the reviewer’s observation that older adults from low socio-economic status continue working if their financial circumstances do not permit them to retire. Those who are higher up in the socio-economic status and do not want to give up earning or those who are passionate about their work may continue working as well. We believe that, while individuals may potentially self-select into informal/formal sector of employment in their young working age, they do not continue to do so at an older age. Although the study sample is skewed towards low education level (78%), it does not include primarily the bottom of the income distribution (low level of wealth) which represents 40.7%, compared to 34.9% and 24.4% in medium and high wealth levels, respectively. Also, the sample information on how long people have been working in their present occupation reveals that, on average, they have been in their present occupation for 36 years, rather than choosing the sector of employment and/or shifting jobs at an older age. Moreover, formal and informal sector work types in India encapsulate a broad range of occupations and, while individuals might select the type of occupation (within the formal or informal sector) due to their pre-existing health status, it is unlikely that they will select between formal and informal sector activities. Having said that, we do not deny the fact that some people with greater physical and cognitive impairment may self-select into one of these sectors from the very beginning of their work life and not at an older age. We assume that would be a minority in our sample. Therefore, we believe that working beyond retirement age due to financial limitations and the nature of work (formal/informal) could influence older people’s health conditions which is the focus of this research.

We are aware of the potential bias due reverse association, as we are limited to use cross-sectional data in the absence of longitudinal data. However, we believe that the use of covariates’ adjustment in the multivariable regression models performed will alleviate both self-selection and reverse association induced bias. Additionally, we have addressed and discussed these issues in the study limitation section (pages 35, lines 15 to 19).

**Reviewer 1 - Comment#2**: Pg 5 of the manuscript: “However, no study till date, nationally or internationally, has emphasised this aspect (concerning the extent to which the health conditions are associated with the type of employment for older workers). I argue that the following reference looks at this type of association, which is not cited in the paper.

Nag, A., Vyas, H., & Nag, P. (2016). Occupational health scenario of Indian informal sector. Industrial health, 2015-0112.

Although they do not specifically focus on the older workers, their sample includes a vast population of workers. Thus, I think that this study should be acknowledged at least.

**Reply:** Thank you for the suggestion.

The paper by Nag et al. (2016) has discussed about occupational hazards and injuries of selected cases of the informal sector. Based on their finding, they have advocated for protective measurement to safeguard the workers from the harsh work environment. This paper is not directly related with our study because we are focusing on long term health conditions. However, we have cited this paper in our discussion section as some of its finding can provide support to our arguments (page 33, line 15; page 34, line 1).

**Reviewer 1-** **Comment#3**: Page 6: in Figure 1, I think another critical piece of information to be taken into account is the initial health endowment: following the seminal contribution of Grossman (1972), health is part of the human capital of each individual; thus, the initial health endowment should be taken into account when evaluating the effect of the type of work on late-life health effects.

For example, the authors should consider including health as a child. To avoid the omitted variable bias problem, I advise the author to include the information about the childhood health conditions available in the LASI questionnaire on questions HT231, HT232, HT233 HT234. This information is much needed to be considered to control for the initial health endowment.

**Reply:** Thank you for the suggestion.

To avoid the omitted variable bias problem, we have incorporated childhood health conditions and the duration of current work in the study as covariates to control for. This is seen in the abstract (page 2, line 9), materials and methods (page 10, lines 12, 22-24) and in Tables 4-7.

**Reviewer 1 -** **Comment#4**: Page 7: There is a reference to the total sample of the survey, but how many individuals are aged above 60? The text-only reports those who are still in the workforce.

Section 2. (page 8) Functional Limitations: there is no reference to which ones are the 13 specific activities

Type of work section (page 9): there is no reference to the complete list of classification of occupation nor in the appendix. Al least an example should be provided to allow the reader to get a grasp of the classification.

**Reply:** Thank you for the comment.

We have added number of older individuals 60 years and above in data source section (page 8, line 7). We have added tables describing functional limitations (table S14) and type of work (table S13) in the appendix section.

**Reviewer 1 - Comment#5**: Methods (page 7 onwards): Although this study looks at the association between type of work and health outcomes using logistic regression models, I wonder why the authors did not provide alternative models such as probabilistic or ols models. In this context, I also argue that the choice of measuring CHC as a function of the polynomial in Model-3 would suggest the need to instrument this variable. Thus, when looking at FL and PCF outcomes, including the CHC as control could be done by exploiting the Control Function approach à la Wooldridge (2015), thus instrumenting the CHC with residuals of Model-3 in Table-1.

Finally, are the results reported in Tables 4 to 6 coefficients or marginal effects? To compare results across Tables, marginal effects should be displayed to have a sense of the magnitude.

**Reply:** Thank you for the comments and suggestions.

1. The reviewer is correct. Alternative models such as probabilistic (probit/tobit) or OLS models can also be used for the analysis. However, in our analysis we wanted to compare those workers who had poor health outcome by their type of economic activity. For example, for cognitive functioning we have compared only those who have poor cognitive level to understand if person is likely to experience PCF, how the risk of PCF varies by type of work. For this purpose, we have constructed our variable in binary form (presence vs. absence) and used logistic regression model.
2. We thank the reviewer for the suggestion regarding CHC residual, it really helped in improving the results of our analysis. We have incorporated CHC residual as a control variable in all our models for Functional Limitations and Poor Cognitive Functioning, instead of using the actual values for CHC (as shown in Table 5, 6 and 7, pages 20-26). The reviewer’s comment also prompted us to consider a new outcome variable which combines both poor cognitive functioning and functional limitations (PCF and/or FL) (page 8, line 19 and page 9, lines 22-24). The new variable is motivated in the research framework section (page 6, lines 23-25, page 7, lines 1-8). The following text has been added into the manuscript.

Page 6, lines 23-25 - *However, the functional limitations and poor cognitive functioning are closely related to each other, as evident from previous studies that physical disabilities or functional limitations increases the risk of poor cognitive functioning in older persons.*

Page 7, lines 1-8 - *Rajan, Hebert (2013), Chodosh, Miller‐Martinez (2010) elaborates that functional limitation plays a key in amplifying the risk of cognitive decline through neurodegenerative processes. Likewise, poor cognitive is associated with high likelihood aggregated functional limitations (66-68). McGuire, Ford (2006) mentioned that older people with lower level of cognitive are more likely to become physically disabled than those with high cognition. Based on the findings of these studies, the combine variable of poor cognitive functioning and/or functional limitation is constructed to provide a better relationship between type of work, physical and cognitive functioning.*

Page 9, lines 22-24 – *The PCF and/or FL variable is constructed by combining Poor Cognitive functioning and Functional limitation health outcomes. Below is description of PCF and/or FL:*

$$PCF\_FL= \left\{ \begin{aligned} 1, if PCF=1 and/or FL=1 \\ 0, otherwise \end{aligned} \right.$$

Results of PCF and/or FL is given below (page 26, lines 2-15; page 27, lines 1-8):

*Table-7 exhibits the relationship between type of work and PCF and/or FL. Result shows that both the type of work and CHC does significantly affect PCF and/or FL in model-1 and model-2. Informal older workers have 1.439 times (p<0.0001) more odds of PCF and/or FL compared to formal older workers. This relationship remains significant after controlling for socio-economic and demographic variables in model-2 but loses its significant level after controlling for lifestyle-behaviour, work characteristics and childhood health in model-3. In case of CHC residual, the odds of PCF and/or FL increase with increase in one unit of CHC residual in model -1. Further, after adding interaction term type of work and CHC residual, the odds of PCF and/or FL among formal older workforce increases with one unit increase in CHC residual (model-2: 1.358, p<0.01; model-3: 1.346, p<0.01). Finally, the interaction term between CHC and type of work is non-significant, and the multiplicative factor estimated through the interaction is close to 1, meaning that the effect of CHC is roughly similar in both formal and informal workers (i.e., no effect modification).*

*Apart from these key results, among all covariates, it appeared that females are more prone to PCF and/or FL, while educated and wealthy are less likely to suffer from the same. Further, high odds of FL are common among those who are engaged in unhealthy lifestyle behaviours such as drinking alcohol, but health lifestyle such as rigorous or moderate physical activity reduces the risk of PCF and/or FL. Besides, the odds of PCF and/or FL are 1.378 (p<0.0001) times greater among those who are not currently married. Finally, across India, the odds of PCF and/or FL are relatively considerable in Eastern, Southern and Western regions compared to North.*

3. Thank you for suggesting marginal effects to display the magnitude of effect of each co-variable, other covariates being held constant. But as far as we believe, odds ratio can also be used to present magnitude of effects of the covariates, like marginal effects do. With the logistic regression approach, we are modelling the risk or probability of the event (e.g., risk or probability of experiencing PCF). The choice of odds ratios is automatic as we are comparing the odds of PCF in formal and informal groups. Regression coefficients are at the log-scale; by taking their exponentiation, we reverse back to the odds scale.

**Reviewer 1 -** **Comment#6**: Table-2: The sample composition is not gender-balanced. This might have affected the results. I also wonder whether the place of residence, which is proportioned toward the rural area, has again some drawbacks such as self-selection of those individuals who are poorer and less educated (in line with the 78% of individuals in the sample reporting low education level).

The role of household size appears to be particularly important in Indian society. I would suggest the author show the distribution of household size greater than 4, given that it includes 61.34% of the sample.

**Reply:** Thank you for the comment.

1. We have cross-checked our result through sensitivity analysis by gender, place of residence and age groups. A section on sensitivity analysis is added in the manuscript (page 29, lines 3-23; page 30, lines 1-3).
2. The figure below shows the distribution of household size greater than 4. The highest of percentage of household size is concentrated between 4 to 8 members of the household. However, we re categorized the household size variable into three categories namely 1. 1-3 members, 2. 4-7 members, 3. 8+ members.

**Reviewer 1 -** **Comment#7**: Bivariate analysis results (page 13): I suggest adding the confidence intervals to Figure 2.

**Reply:**  We thank the reviewer for this useful comment. The confidence intervals have now been added in the figure 2.

**Reviewer 1 -** **Comment#8**: Tables from 4 (page 15): as a general comment, I wonder why doing yoga could increase the chances of CHC and how the authors explain this finding.

**Reply:** Thank you for the comment.

Thank you for pointing out this result. The increase in CHC using Yoga is an interesting result because we are using cross-sectional data and can only justify association. So, it could be possible that, those people who are performing yoga, are more likely to be suffering from any chronic health conditions.

**Reviewer 1 -** **Comment#9**: As a general comment, I would suggest the authors conduct sensitivity analyses by subgroups of individuals, namely by gender, geographical area, and age group 60-64 and 65+. This could confirm the robustness of the results.

Another alternative classification related to formal vs informal jobs is the separation between non-strenuous vs strenuous jobs. This could be investigated as an alternative.

**Reply:** We thank the reviewer for this insightful comment. We believe that addressing this comment in the revised draft will improve the clarity of the results.

As mentioned in the previous comment (comment #6), the sensitivity analyses have been performed by gender, geographical area, and age groups.

The sensitivity analysis has confirmed the robustness of our result, which is described below (page29-30):

1. *For CHC - the effect of type of work is significant only in male (Table S1) and 60-65 age group (Table S3) with formal workers having higher odds of CHC.*
2. *For FL - the type of work, without CHC residual (CHC residual=0), exhibit significant effects on FL (less odds of FL for informal compared to formal workers) within females (Table S4) and 65 + age group (Table S6) only. Moreover, the effect of CHC is much stronger among informal male workers (OR=1.585) than female workers (OR=1.412), and even much stronger among informal urban workers (OR=1.857) than rural workers (OR=1.449), while within age groups, the effect is much stronger among formal 60-65 aged workers (OR=1.667) than 65+ aged workers (OR=1.340) (Tables S4 to S6).*
3. *For PCF - informal workers exhibit higher odds of PCF within both male and female, rural and urban areas, and age groups. However, the effects of type of work are significant only within male (Table S7), rural area (Table S8) and 65+ age group (Table S9) workers.*
4. *For PCF and/or FL - The effect of CHC is significant within male and female, although slightly stronger among informal male workers (OR=1.370) than formal male workers (OR=1.334) (Table S10). Moreover, the effect of CHC is also significant within 60-65 age group only, but much stronger among formal workers (OR=1.452) than informal workers (OR=1.365) (Table S12). Finally, the effect of CHC is also significant within both rural and urban residence areas, but much stronger among urban workers (informal: OR=1.653; formal: OR=1.449) (Tables S11).*

Thank you for suggesting an alternative classification for the type of work, which we believe would provide a different insight on the association between the type of work and health outcome. This suggestion can be examined for our future research because analysis based on non-strenuous vs strenuous job classification in the present paper would deviate from our study objective which focuses on the classification based on two major economic activities.

Minor Points to be addressed:

**Reviewer 1 -** **Comment#10**: Page 3: When reporting “India, …., reportedly 8.6 percent (104 million) of the total population in 2011…” I think a reference should be added.

Page 6: Why there is the name of the authors reported next to reference number (7)? I think that the manuscript should show consistency with the style of referencing.

**Reply:** Thank you for the comment.

1. Reference has been added in this statement.
2. All the referencing errors have been corrected.

***Reviewer 2:***

**Reviewer 2 - Comment#1**: My major comments are on the complexity of employment in later years as established by existing research (e.g., Rietveld et al.,2014; van Zon et al.,2020). It is understandable that the cross-sectional nature of the dataset limits further exploration of any dynamic aspects of employment, also, the goal of the paper is not aimed at a causal identification. However, it could be more informative to add some relevant descriptive statistics, sensitivity checks, or comments regarding the defined employment status that might not truly reflect the general associations.

1) The observed employment type might contain some noise due to potential complex work transitions in later years such as partial retirement. Some transitions and jobs could be quite recent and temporary, especially in the informal sector, which may require additional control for working characteristics such as working years and sectors (public/private).

Without any further details, it is possible that some people are working as a recovery from an early retirement or mandatory retirement, or at a phase of bridge job. For one thing, the current employment can differ much from their main career which determinants one’s pension and other welfare schemes.

For another, the health effect of employment might be biased given potentially complex sorting into employment for older workers. For instance, if one transited from a full-time office-based job to self-employment to better manage working hours, the estimated health effect of the informal sector might vary by time. Socioeconomically advantaged individuals might extend working life as a way to continue social participation which probably exerts a positive impact on mental health and well-being, in the meanwhile, affecting physical health.

**Reply:** We thank the reviewer for this insightful comment.

1. We agree with reviewer that past work characteristics and job transition are important indicators that could have been useful in our analysis, however LASI data does not provide information pertaining to past jobs. Further, as per the Indian context, the phenomena of job transition are quite unlikely, especially a shift from formal to informal sector are not common. As per our analysis, 64% of the sample participants spent 30 years or more in their current job activity, and on average, people spent 36 years in their present job. For older population (age of 60 years and above), these statistics indicate low level of job transition. Moreover, in LASI data, public/private sector related information is only applicable for few selected formal sectors (service or salaried person).
2. Without any further details, it is possible that some people are working as a recovery from an early retirement or mandatory retirement, or at a phase of bridge job. For one thing, the current employment can differ much from their main career which determinants one’s pension and other welfare schemes. We agree with the reviewer that the current employment can differ much from the main career. This might be the case for the 19% of sample participants whose duration in the current work is less than 15 years. However, in the Indian context, it is less likely that these people would be working as a recovery from retirement or a phase of bridge job. We have now cross-checked our results through sensitivity analysis by gender, age-group, and place of residence. We believe this added value to the overall presentation of the manuscript and improved clarity of our findings. We have now added this information in the appendix section from table S1-S12. From the sensitivity analysis we have found that the risk of CHC is significantly low among informal male and 60-65 age group workers. Moreover, FL is less prevalent among informal female and 65+ age group workers. Conversely, the risk of PCF is relatively high among informal male, rural areas, and 65+ age group working population.

**Reviewer 2 - Comment#2**: It would be better to add separate estimation by gender considering the gender difference in terms of both health, healthcare utilisation and employment aspects (e.g., Kandrack et al.,1991). Especially, labour force participation is more selective among females than males, and there are also great heterogeneities across occupations within formal or informal sectors. For instance, continued employment may buffer against risk factors that aggravate women’s cognitive health (Oi, Katsuya 2019).

**Reply:** Thank you for the comment.

We thank the reviewer and agree that gender specific analysis will add value to this research. We have now conducted separate gender specific analysis. Please refer to our response to your Comment#1 and Reviewer-1’s comment#9. While the objective of this research is to study the association between type of work and health outcomes. We will study, healthcare utilization by type of work in the next research objective (as part of PhD thesis) which will be covered in a different paper.

***For other minor comments:***

**Reviewer 2 -** **Comment#3**: It might be better to cluster standard error at a household level to account for the potential correlation among employment/retirement decisions within a household. Old couples might have a joint retirement (or working) plan.

**Reply:** Thank you for the comment. While we agree with the reviewer’s concern about cluster standard error at household level as it might be affecting the consistency of the coefficient estimates. However, we believe that might not be a major issue in this study as around 80% (from LASI data) of the respondents in our sample are single working members from a household.

**Reviewer 2 - Comment#4**: The implication about the healthcare services needs could probably be better supported by adding some descriptive statistics about healthcare utilisation, and healthcare entitlements (if data permits).

**Reply:** Thank you for the comment.

We agree with the reviewer’s concerns on health service utilisation, and we are addressing that in another study as part of the PhD thesis.

**Reviewer 2 - Comment#5**: Potential measurement error due to cognitive impairment. To what extent the estimated might be affected by the recall error in covariates such as wealth and job characteristics? Any potential screening criteria for cognitive impairment? Are surveyed people all self-interviewed or include proxy answers?

**Reply:** Thank you for the comment.

In LASI, the Health and Retirement study (HRS) module has been used to derive cognitive measures. These measures are collected using various domains- including memory, orientation, retrieval fluency (verbal fluency), arithmetic, executive functioning, and object naming. To measure the cognitive functioning, the respondents were asked certain questions pertaining to the cognitive measure domains and the responses were noted by interviewer based on the feedback or reply from the respondents, so no proxy person was involved during the interview. Further, as per the LASI report, those participants were excluded from the measurement who sought or receive assistance during interview.

**Reviewer 2 - - Comment#6**: Typos:

P17: informal workers without CHC have 0.8 folds less odds

P26: those determinants which have considerable impacts

**Reply:** Thank you for the comment.

All the typo errors have been rectified in the manuscript.
