# Supplementary material for: Informal sector employment and the health outcomes of older workers in India
Source: PLoS One. 2023 Feb 22;18(2):e0266576. doi: 10.1371/journal.pone.0266576 (PMC9946227; doi:10.1371/journal.pone.0266576)
Supplement: Attachment — Submitted filename: Reviewers reply_final.docx [file pone.0266576.s004.docx]

We would like to thank the reviewers for their useful comments. Please consider our responses below. Please note that all references to line numbers in this response letter relate to line numbers in the document with track changes.

**Reviewer reports:**

**Reviewer 1- Comment#1:** I think the authors have responded satisfactorily to most of the comments from the first review round. I do however still have some small comments for some of them. In particular, I think the role of individuals with poor health self-selecting into poorer jobs, which leads to a stronger deterioration of health should be acknowledged better than as it is now in the manuscript. Although it is unlikely that individuals self-selected themselves into the type of employment due to pre-existing health status later in life, it could be that they self-select at the beginning of their working careers and they remain in poor jobs. It is fundamental to stress that individuals could ex ante engage in poor jobs due to their precarious health status at a young age and thus leading to late-life health decline. I think this point could be better explained in the introduction, as they explain in the report which I received.

**Reply:** We thank the reviewer for this useful comment.

As per the reviewer’s suggestion, we have added some lines on “self-section into job” in the research framework section of manuscript (page no. 6 and line no. 13-21).

The paragraph is as follows:

“However, the relationship between type of work and health could also be affected by self-selection bias in which a person may self-select into the type of work due to pre-existing health conditions, even from younger age. Meaning that there may be two-way relationship where the pre-existing health conditions influencing the type of employment and vice versa. In the case of India, the self-section into poor jobs is implausible because formal and informal sector types of work encapsulate a broad range of occupations. While individuals might select the type of occupation (within the formal or informal sector) due to their pre-existing health status, it is unlikely that they will select between formal and informal sector activities and continue to do so at an older age.”

**Reviewer 1*-* Comment#2:** Finally, regarding the Discussion session, I found the section very dense and I would suggest stressing the key findings of the paper as well as the take-home message, in order to provide a more synthetic interpretation.

**Reply:** Thank you for pointing this out, we have improved our discussion section by focusing only on the key findings of the paper, and we have also removed some paragraphs which were not that important. Further, discussion section is segregated in 5 aspects: 1. basic scenario of older workers in India, 2. association between type of work and health outcomes, 3. role of other covariates, 4. sensitivity analysis, and 5. study limitations.

Following paragraphs, we have removed from the discussion section:

1. From page no. 29, line 10-17

Consequently, agrarian households tend to have larger family size which is assumed to provide better manpower to support farming activities. This can be clearly seen through current findings showing higher work participation for larger family size households. However, it is also important to note that the level of engagement in informal activities is considerable for those households with small family sizes. Tafuro (2020) elucidated that intergenerational support is strongly related with son’s preference in countries like, India, China, Vietnam, and South Korea, and it is quite common that older people without sons are more likely to be economically active in these regions.

2. Page 31 (line 24-25) and page 32 (line 1-2)

It is because Japan is a developed country where provision for social and financial security is strong, therefore older workers only engage to maintain their social network and get the sense of pride of being economically independent.

3. Page no. 32, line 13-19

Heterogeneity in context of health outcomes across regions can be clearly observed in India. Notably, chances of CHC upsurges in southern region, but FL is relatively intensifying in southern and western region. Geographically, there is a stark difference in terms of PCF, it rises in western region, then dwindles in north-eastern region. Further, the combine conditions of PCF and/or FL are high in eastern, western, and southern regions of India. Despite of physical and mental health limitations, older population in India are still working after the retirement age especially in informal sector.

**Reviewer 1*-* minor comment#1:** Table 2: I wonder whether for binary covariates it would be sufficient to report only one category (for example only those who have chronic health conditions, instead of both no/yes), to make the tables more compact.

**Reply:** Thank you for the suggestion.

Now, we have reported only the percentage of health outcomes variables, and other binary covariates in Table-2 (page no.13).

**Reviewer 2*-* Comment#1:** Many thanks for your efforts in the revision, and I think all my concerns have been responded or addressed properly.

For the new version, I might have two additional comments. The first is the control function approach which has changed many models. If I understand correctly, it is used as a control for partial chronic conditions that are unexplained by observables. If much of variation in chronic conditions have been explained in a separate model, the residual variable might not be a good proxy for chronic conditions.

**Reply:** Yes, CHC residual is the unexplained partial chronic conditions which we have calculated through the following procedure:

1. We have run the full model for CHC incorporating all the covariates (Table-4, model-3).
2. Then, the predicted value of CHC is calculated.
3. Further, the residual value is calculated by subtracting predicted CHC from the observed CHC.

So, the reviewer’s understanding is correct, if most of the variation is explained in chronic conditions, taking residual variable of chronic condition should not be a good proxy. However, in our case, the CHC residual in Table-5, Table-6, and Table-7 for model-1 is coming significant, which means that there are still some unexplained variations in CHC which is also influencing our outcome variables. Therefore, we could say that taking CHC residual as a proxy for CHC is a better option. Moreover, the use of the control function was suggested by the first reviewer in the first review round. Indeed, the reviewer #1 stated: “Thus, when looking at FL and PCF outcomes, including the CHC as control could be done by exploiting the Control Function approach à la Wooldridge (2015), thus instrumenting the CHC with residuals of Model-3 in Table-1”.

**Reviewer 2*-* Comment#2:** Second, I am not sure whether I grasp the idea of combining PCF and FL as the fourth outcome variable to explore the potential interactions between PCF and FL. Indeed, in the final results, the coefficients of informal worker on PCF and FL have opposite signs, making the coefficient on PCF/PF insignificant. I am not sure about the idea here or the relevant explanation.

**Reply:** Thank you for the comment.

The rationale behind combining PCF and/or FL is constructed to search for a better and stronger relationship between type of work, physical and cognitive functioning. Further, previous literatures have emphasised that PCF and FL are the two-sides of the same coin because functional limitation plays a key in amplifying the risk of cognitive decline (Miller Martinez et al., 2010; Rajan et al., 2013), whereas poor cognitive is associated with high likelihood of functional limitations (McConnell et al., 2002; Dodge et al., 2005; McGuire et al., 2006).

The coefficients of informal worker on FL, PCF, and PCF/FL (main effects, Model 1 in Tables 5, 6 & 7) have all positive signs and are statistically significant. The opposite signs for coefficients of informal worker on PCF and FL in the final model results should not be interpreted as the main effects since these models include the interaction term type of work * residual CHC. These coefficients represent the effect of type of work in the absence of any residual CHC (i.e., residual CHC = 0). Moreover, the final models have been adjusted for various covariates, some being enough important to explain some of the variation in FL, PCF, and PCF/FL. The insignificant result of type of work on PCF and/or FL may not necessarily due to the opposite signs but to other covariates controlled for in the model. It may suggest as well that the type of work could not have any influence if a person suffers from both the conditions. Further, other unhealthy life-style behaviours may play an important role in distorting the relationship between type of work and PCF and/or FL. The rationale of PCF and/or FL and reason for insignificant relationship between type of work and PCF and/or FL is already mentioned in research framework section (page no. 7 and line no. 5-15) and discussion section (page no. 30 and line no. 14-17).

**Research framework (page no. 7 and line no. 5-15):**

However, the functional limitations and poor cognitive functioning are closely related to each other, as evident from previous studies which reported that physical disabilities or functional limitations increase the risk of poor cognitive functioning in older persons. Indeed, Rajan, Hebert (2013), Chodosh, Miller‐Martinez (2010) elaborates that functional limitation plays a key in amplifying the risk of cognitive decline through neurodegenerative processes. Likewise, poor cognitive is associated with high likelihood aggregated functional limitations [66-68]. McGuire, Ford (2006) mentioned that older people with lower level of cognitive are more likely to become physically disabled than those with high cognition. Based on the findings of these studies, the combined variable of poor cognitive functioning and/or functional limitation is constructed to search for a stronger relationship between type of work, physical and cognitive functioning.

**Discussion (page no. 30 and line no. 14-17):**

However, this relationship is not significant after controlling for work-characteristics, lifestyle behaviour and childhood health conditions. The major factor which distorts the relationship between type of work and PCF and/or FL is unhealthy lifestyle behaviour namely such as drinking alcohol.
