# Supplementary material for: Informal sector employment and the health outcomes of older workers in India
Source: PLoS One. 2023 Feb 22;18(2):e0266576. doi: 10.1371/journal.pone.0266576 (PMC9946227; doi:10.1371/journal.pone.0266576)
Supplement: Attachment — Submitted filename: response_to_reviewer_3rd.docx [file pone.0266576.s005.docx]

We would like to thank the reviewers for their useful comments. Please consider our responses below.

**Reviewer reports:**

***Reviewer 1:***

The authors have addressed all my comments. However, I want to stress that the discussion needs some adjustments. The discussion should focus on the main findings and the paper's strength and limitations, as well as potential future research. As it is now the discussion includes also the basic scenario (which is well explained in the introduction) and the role of covariates (which I do not find essential in this section). I think the authors could improve the discussion. After that, the paper would likely be ready to be accepted for publication.

**Reply:** We thank the reviewer for this useful comment. As per the suggestion of the reviewer, we have removed the points pertaining to roles of covariates from the discussion as the interpretation of other covariates is already given in result section. Following are the points which we have removed from page no. 30 (lines 8-25) to 31 (lines 1-21).

“This research also sheds light on those determinants of unfavourable health outcomes which are socio-economic and demographic attributes, work characteristics, lifestyle behaviour, childhood health and regions of India. Among them, prolonged work engagement has detrimental effect on physical and cognitive functioning for those who are female older workers if likened to male counterparts. Commonly mainstream female workers are employed in informal activities, and they alone work as domestic workers giving rise to double burden of activities [51, 52, 78]. Additionally, it is known that women live longer than men and continue to work in their later life with one or more disabilities [11, 52, 79]. Increasing age has adverse implications on health conditions of older people [22, 80, 81], which can be observed through the result that 65+ working population tend to suffer more from physical and mental health conditions. In Indian society, ST and Muslim community are also the part of vulnerable and marginalized sections who tend to work longer in later ages. The risk of PCF is substantial among ST groups, while the burden of CHC is more prominent among Muslim community. This is may be because ST group is mostly engaged in manual labour or scavenging [82], and agricultural labour activities [83] which is associated with decline in cognitive ability [51]. On the other hand, Muslim community in India are well known for their engagement in informal artisanal work such as weaving, carpentry, black-smithing, and Zari work which is related ergonomic condition leading to CHC [78, 84-87]. However, the combination of PCF and/or FL is remarkably high among both ST group and Muslim community which reflects having these poor conditions altogether elevates the health risk for any marginalized community as compared to their well-off counterparts.

Education and wealth are considerable predictors of unfavourable health outcomes. Increase in education and wealth leads to reduction in FL, PCF, and PCF and/or FL, though escalating the chances of CHC. As evident from studies that better education and wealth improves the physical functioning and work ability [44, 45]. In case of work characteristics, increase in working hours and wages is associated with improvement in PCF, but this result is contradictory to the studies conducted in Japan which states that part time or low working hours improves the cognitive functioning of older workers [10, 11]. Although as it is cross-sectional study, the results could also reflect that only those older people are working longer who have better cognitive functioning. The life-style behaviours are essential components which needs to be taken into account to justify the relationship between type of work and health. Drinking alcohol and consuming tobacco/ smoking can lead to serious health implication, as evident from the result that it degrades physical as well as cognitive functioning. Subsequently, vigorous physical exercise diminishes the risk of CHC and FL, however Yoga/ Pranayam ameliorates cognitive functioning. Moreover, adverse impacts of CHC and FL can be minimized by staying active and maintaining a healthy lifestyle [88-90]. On the other hand, poor childhood health condition worsens the risk of CHC, and FL. Similar findings can be seen in the findings of Pavela and Latham (2016), White, Campo (2013) that poor childhood conditions are strongly associated with development of chronic conditions in later ages.”

Moreover, we have added few lines related to potential future research in page no.32 and line no. 8 to 11.

“Thus, future studies based on cause-effect relationship between type of work and health outcomes would provide a platform for preventive strategies to deal with health-related issues of working older population.”

***Reviewer 2:***

Many thanks for the updated version. All my comments have been explained in general. My small concern will still be around the justification to combine PCF and FL although they are closely and positively correlated. For me, the separate results for each as outcome variable seem to be already informative about the effect of informal sector on health outcomes. The mechanisms might be different for cognition and functional limitation.

**Reply:** Thank you for pointing out your concern pertaining to combined variable of PCF and FL. Our rationale of PCF and/or FL is based on the previous literatures which have emphasised that PCF and FL are the two-sides of a same coin as both are related to each other. Further, we were also interested to see the influence of type of work if a person is having both the health conditions. Indeed, separate analysis have provided the informative findings, but we have also found that type of work does not play a significant when a person suffers from both the conditions. In other words, having PCF and FL together could diminishes the influence of type of work.
